# Health-related quality of life and mental distress in patients with partial deafness: preliminary findings

**DOI:** 10.1007/s00405-015-3713-7

**Published:** 2015-08-05

**Authors:** Katarzyna Cieśla, Monika Lewandowska, Henryk Skarżyński

**Affiliations:** Institute of Physiology and Pathology of Hearing, World Hearing Center, Mokra 17, 05-830 Warsaw/Kajetany, Poland; Centre for Modern Interdisciplinary Technologies, Nicolaus Copernicus University in Torun, Torun, Poland

**Keywords:** Mental distress, Health-related quality of life, Partial deafness, Postlingual hearing impairment, Prelingual hearing impairment

## Abstract

The aim of the study was to evaluate mental distress and health-related quality of life in patients with bilateral partial deafness (high-frequency sensorineural hearing loss) before cochlear implantation, with respect to their audiological performance and time of onset of the hearing impairment. Thirty-one patients and 31 normal-hearing individuals were administered the Beck Depression Inventory (BDI), the State-Trait-Anxiety-Inventory (STAI) and the World Health Organization Quality of Life-BREF questionnaire (WHOQOL-BREF). Patients also completed the Nijmegen-Cochlear-Implant-Questionnaire (NCIQ), a tool for evaluation of quality of life related to hearing loss. Patients revealed increased depressive and anxiety symptoms, as well as decreased health-related quality of life (psychological health, physical health), in comparison with their healthy counterparts (*t* tests, *p* < 0.05). Furthermore, a General Linear Model demonstrated in patients with a prelingual onset of hearing loss enhanced self-evaluated social interactions and activity (NCIQ), when their outcomes were contrasted with those obtained in individuals with postlingual partial deafness (*p* < 0.05). The study failed to show any effect of collateral tinnitus. Patients not using hearing aids had better audiological performance and, therefore, better sound perception and speech production, as measured with NCIQ. There was no effect of hearing aid use with respect to mental distress. Additional statistically significant correlations seen in patients included those between a steeper slope hearing loss configuration (averaged pure-tone thresholds at 1 and 2 kHz with subtracted threshold at 0.5 kHz) and better audiometric speech detection, between audiometric thresholds and the subjectively rated sound perception (NCIQ), as well as left-ear audiometric word recognition scores and the subjectively perceived ability to recognize advanced sounds (NCIQ). In addition, a longer duration of postlingual deafness, as well as a younger age at the onset were both related to worse speech detection thresholds. The results of the study provide evidence that successful rehabilitation in patients with partial deafness might have to go beyond the standard speech therapy. Enhancement of the regular diagnostic assessment with additional psychological tools is highly recommended. Further investigation is required as to the role of functional residual hearing, hearing aid use and tinnitus, in relation to future outcomes of cochlear implantation.

## Introduction

A hearing impairment is not only a disability (a communication dysfunction) but can also be perceived by an individual as a handicap with its psychosocial effects. Patients often encounter confusion, stigmatization or even mockery. The extent of the handicap, however, cannot be predicted from the audiometric profile itself. It has been argued that behavioral and affective variables have to be considered to provide successful management of the disease. Consequently, new tools are being introduced to clinical practice measuring health-related quality of life (HRQoL) in patients, including its core element, mental health, along specific psychological tools to assess psychopathology (mental distress). Still, however, it remains extremely challenging to capture the non-tangible psychosocial aspects of hearing loss and thereby predict communication and adjustment hardships of patients, as well as their potential benefit from treatment and rehabilitation with e.g. cochlear implantation [1–5].

Whereas patients with a postlingual onset of deafness grow up with a hearing identity to suddenly or progressively be devoid of the auditory sense, those born with a hearing impairment are never exposed to a non-degraded acoustic and speech surrounding. Some authors suggest that underdeveloped communication skills at an early age can deteriorate emotional and social development (and potentially also neurological), with others arguing that an altered identity from hearing to deaf in a later-onset deafness can actually be more detrimental to mental health (see Ohre and colleagues for a review [4]).

Several large- and medium-population studies have indicated increased mental distress among patients with an acquired postlingual hearing impairment (with an onset after developing language skills), as compared to the general population. Depressive/anxiety symptoms and social isolation were found most distinctive [1–8]. Findings concerning the correlation between audiological measures, such as pure-tone audiometry, and mental health have been contradictory, probably since numerous factors can contribute to the development of a mental distress and a sensory impairment can be one of those [4]. Thomas and colleagues reported a four times larger scoring above cut-off for significant anxiety/depression symptoms among patients with a hearing impairment than in the general population, with the proportion twice as large for a deficit of 70 dB and above [8]. At the same time, two example studies revealed no clear association between the objectively measured hearing-loss severity (acquired, moderate to profound) and the frequency of depressive symptoms [1, 2]. It was rather the individual attitude towards the disability, as well as their coping strategies that were indicated as major predictors of the psychological well-being [2]. In addition, these and other trials provided evidence of annoying tinnitus as a factor increasing the depressive mood in patients [1, 2, 9, 10]. Lower energy levels, greater distress and social isolation were also found in the patient population using HRQoL tools, with again none of the objective audiological measures consistently indicative of the individual quality of life [7]. Hallam and colleagues suggested that mental health in the hearing impaired was affected by the self-assessed level of communication skills, self-esteem and acceptance of the disability, as well as coexisting medical conditions [3; cf. 7]. Both trials, furthermore, showed lower HRQoL in women, with contradictory findings reported as to the predictive value of satisfaction with hearing devices [3, 7].

Increased mental distress, and especially elevated anxiety, depression and interpersonal sensitivity have also been detected in the profoundly deaf population using sign language [3, 11, 12]. These trials required specifically designed assessment tools adapted to sign language [13]. In a study by Hallam and colleagues there were no specific effects demonstrated of audiological variables on psychopathology levels, except for a comorbid medical condition in patients with prelingual severe to profound deafness. Furthermore, in terms of the health-related quality of life, neither presence of tinnitus nor satisfaction with hearing devices was found a predictive factor. As was the case of the co-studied population of patients with postlingual hearing deficits, the scores in signing patients deteriorated with poorer acceptance of the disability, as well as among women [3]. The sex effect on HRQoL was further confirmed by Fellinger and colleagues (in this study women were also reported to have more significant depressive symptoms) [11]. Tinnitus was either found to have no effect on the quality of life of patients with a prelingual hearing loss or this comorbidity was an uncontrolled variable [3, 11, 12].

Partial deafness is a special type of sensorineural hearing loss, with a severe to profound impairment at frequencies above 1–2 kHz and normal to moderately deteriorated hearing acuity at lower frequency bands [14, 15]. With preservation of relatively good audio-oral communication and support from lip-reading, noisy and multi-talker situations still remain very challenging for this population. Therefore, one suggested and successful treatment option for partial deafness is cochlear implantation (CI), including a combination of a cochlear implant and a hearing aid in one ear (electroacoustic system, EAS) [15, 16]. The Institute of Physiology and Pathology of Hearing (Warsaw, Poland) has a long tradition of providing pre- and postoperative medical and psychological services to patients with various subtypes of hearing deficits, including cochlear implantation in partial deafness [14, 17]. This is a preliminary study investigating health-related quality of life, as well as the prevalence of psychopathological symptoms in patients with residual hearing on low frequencies. All patients will participate in a follow-up visit involving an identical diagnostic assessment after at least 6 months of cochlear implant use. The authors seek to explore various relationships between audiological, demographic and psychological measures which might in the future be investigated as predictors for CI-outcomes. Pre- and post-implantation outcomes will be compared to appraise, among others, the improvement of HRQoL after the intervention [5, 18–20].

## Materials and methods

### Participants

**Fig. 1:**
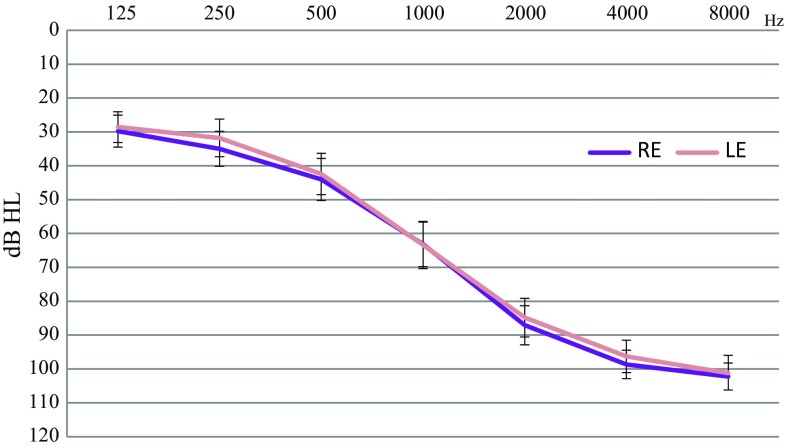
Group mean air-conduction pure-tone audiometry results for the right ear (RE) and the left ear (LE), with *bars* indicating standard deviations

Thirty-one patients (16F, 15M) with a bilateral symmetrical sensorineural hearing loss (partial deafness, hereafter: PD) participated in the study. Patients were all recruited from among a large pool of patients of the Institute of Physiology and Pathology of Hearing in Warsaw, Poland. Some of the patients were already candidates for cochlear implantation at the time of the study. The mean age of patients was 37.6 ± 7.9 years (M ± SD) (age range 18.5–53.8 years) (see Table 1). Only patients under the age of 50 (except for one) were included in the trial, in order to exclude the potential effect of hearing deterioration due to age (such as, presbyacusis). Figure 1 depicts group average air-conduction pure-tone audiometry outcomes. The between-ear difference in PTA (pure-tone average for 0.5, 1 and 2 kHz) was below 15 dB [PTA left vs. PTA right: *t*(30) = 0.96; *p* = 0.34]. Nineteen patients were regular users of either one (10 patients) or two optimally fitted hearing devices (9 patients) and 12 patients were non-users (as they had no significant gain). Sixteen patients had chronic non-bothersome bilateral tinnitus. There were 14 patients with a prelingual hearing loss (developed and diagnosed before the age of 3 years; hereafter: PRE) and 17 patients with a postlingual hearing loss (developed and diagnosed after the age of 12 years; hereafter: POST). All patients had well developed verbal skills and used auditory-verbal communication. In all patients audiometric thresholds were measured for frequencies 0.25–8 kHz. PTA values were then calculated for both ears, using averaged thresholds for 0.5, 1 and 2 kHz. Slope was estimated by subtracting the 0.5 kHz threshold from an averaged threshold for 1 and 2 kHz for each ear separately (a simplified algorithm suggested in Hornsby and colleagues [21]). Patients had unaided speech audiometry examination using a Polish monosyllable word test [22]. The outcomes were Speech Detection Threshold (SDT), i.e. intensity level at which the patient was able to detect speech items (dB), and Word Recognition Score (WRS), i.e. the maximum percentage of the recognized word pairs. All patients’ clinical details and outcomes of comparisons between PRE and POST patient groups were depicted in Table 2. The control group consisted of 31 individuals with normal hearing (hereafter: NH; 16F, 15M, mean age: 34.4 ± SD years = 5.8, age range 26.2–45.2 years). As shown in Table 1, the normal hearing and the patient group did not differ in terms of basic demographic variables (non-parametric statistical tests were applied due to unequal group sizes). All study participants had no history of neurological/psychiatric diseases or any other serious illnesses, nor did they use drugs affecting the central nervous system. All individuals provided written informed consent to participate in the study after all study details had been fully explained. The study was approved by the Ethical Committee of the Institute of Physiology and Pathology of Hearing and was in accordance with the Declaration of Helsinki.

**Table 1:** Demographic profile of patients with partial deafness and normal hearing individuals; outcomes of between-group comparisons (Chi2, non-parametric median tests)

	Postlingual PD (*N* = 17)	Prelingual PD (*N* = 14)	Normal hearing (*N* = 31)	Postlingual PD vs. prelingual PD (Chi^2^/*t*)	Partial deafness vs. normal hearing (Chi^2^/*t*)
Female:male	11:6	5:9	16:15	0.27	0.00
Age (years) Median (minimum–maximum)	40.3 (30.8–53.8)	35.1 (18.5–48.8)	32.2 (26.2–45.2)	1.97	1.81
Education level (subjects)					
Primary school	1	2	0	0.23	3.91
Middle school	7	7	12		
High school	9	5	19		

*PD* partial deafness

* Statistically significant at *p* < 0.05

**Table 2:** Clinical profile of patients with partial deafness; outcomes of comparisons (Chi^2^, *t* tests) between POST and PRE patients

	Postlingual PD (*N* = 17)		Prelingual PD (*N* = 14)		Between-group comparisons *Chi* ^2^ */t*
Duration of HL (years)	16.9 (10.0) (3-40)		NA		NA
Age at onset of HL (years)	24.5 (9.8) (12-49)		NA		NA
Etiology (subjects)					
Idiopathic	16		9		4.92
Ototoxic	1		5		
Bilateral tinnitus (subjects)					
Yes	10		6		0.78
No	7		8		
Duration of tinnitus (years) M (SD)	12.7 (8.6) (*N* = 10)		16.7 (17.3) (*N* = 6)		0.62
No of HAs					
none	9		3		0.15
1	5		5		
2	3		6		
Duration of HA use (years) M (SD)	15.5 (6.1) (*N* = 8)		19.7 (8.7) (*N* = 11)		1.25

	*M* (SD) (range)	Comparisons between ears *t*	*M* (SD) (range)	Comparisons between ears *t*	
PTA R (dB)	58.2 (21.6) (23–97)	1.18	71.5 (18.9) (30–97)	0.27	1.84
PTA L (dB)	56.4 (20.6) (20–83)		70.9 (20.8) (23–95)		1.94
Slope R (dB)	47.8 (26.6) (0–100)	0.1	50.6 (25.7) (20–98)	0.09	0.27
Slope L (dB)	47.5 (24.8) (3–93)		50.1 (24.4) (20–95)		0.29
SDT R (dB)	56.5 (22.5) (15–90)	1.05	70.0 (18.8) (40–100)	0.24	1.82
SDT L (dB)	57.9 (24.3) (10–90)		69.3 (15.8) (40–90)		1.56
WRS R (%)	66.8 (23.8) (25–100)	1.6	51.0 (31.9) (5–100)	0.21	1.52
WRS L (%)	58.2 (27.7) (20–100)		51.8 (26.1) (10–90)		0.66

*PD* partial deafness, *HA* hearing aid, *PTA* pure-tone average, *SDT* speech detection threshold, *WRS* word recognition score, *M* mean, *SD* standard deviation, *L* left ear, *R* right ear, *NA* not applicable

* Statistically significant at *p* < 0.05

### Data collection

First, all patients participated in a comprehensive medical interview and an otolaryngological examination performed by an ENT-specialist at the Institute of Physiology and Pathology of Hearing in Warsaw, Poland. Patients were asked about details of their hearing impairment, including comorbidities such as tinnitus, as well as use of and satisfaction with hearing aids. All patients using hearing aids were satisfied with the fitting at the time of the study. Next, audiometric tests were performed in a sound-proof booth to assess air-conduction pure-tone thresholds and speech recognition outcomes. Experimental Polish versions of psychological questionnaires were administered to participants on the same day during a professional face-to-face psychological consultation. The administration order was randomized among subjects. To assess the prevalence of depressive symptoms among patients and the normal hearing individuals, Beck Depression Inventory (BDI) was administered. BDI is a multiple-choice self-report inventory with 21 questions responded to on a 0–3 point scale, with higher scores reflecting higher severity of symptoms. The maximum score is 63 [23]. State-Trait-Anxiety-Inventory (STAI) Form X was used to evaluate anxiety symptoms in patients and in the control group. The questionnaire comprises of 40 questions divided to two scales, with 20 questions referring to anxiety as a state and 20 evaluating the level of anxiety as a personal trait. The tool is a self-report assessed on a 4-point Likert-type scale (scores 1–4) and higher scores indicate higher intensity of symptoms. The maximum scale score is 80 [24]. Next, patients and the normal hearing subjects completed the World Health Organization’s Brief Quality of Life questionnaire (WHOQOL-BREF), a worldwide-recognized tool to evaluate health-related quality of life. WHOQOL-BREF consists of 4 subscales: physical health (7 questions, max 35 points), psychological health (6 questions, max 30 points*)*, social relationships (3 questions, max 15 points), and environment (8 questions, max 40 points), and includes 26 items in total. A 5-point Likert-type scale is used to provide answers to single questions [25]. Finally, patients were administered the Nijmegen-Cochlear-Implant-Questionnaire [19]. The tool has proven useful in longitudinal assessment of hearing-loss-related quality of life before and after cochlear implantation with relatively good consistency across subdomains, test–retest coefficients and responsiveness indices [19, 20, 26, 27]. Six QoL subdomains included in the inventory are: basic sound perception (phone ringing, steps, street noise, radio, etc.), advanced sound perception (recognizing speech in various acoustic situations, music appraisal, prosody, talking on the phone), speech production (e.g. modulation of voice and intonation) (physical scale), activity and social interactions (social scale), and self-esteem (psychological scale). There are 60 items in the questionnaire which the patient responds to on a 6-point Likert scale. The maximum score in each scale (10 questions) is 50. The psychological assessment took ~1.5 h in total. Patients completed all the written questionnaires on their own.

### Data analysis

To assess the effect of partial deafness on the quality of life and mental distress, a comparative between-group analysis using a two-sample *t* test was performed. Results obtained in patients (POST and PRE were pooled together) were compared with those of normal hearing individuals (BDI, STAI, WHOQOL-BREF). Next, scores of the two subgroups of patients with different onsets of hearing deprivation (PRE vs. POST) were calculated and contrasted with one another, using a Multivariate General Linear Model (GLM). This approach was justified due to the limited sizes of the compared subgroups. The type of the onset of the hearing impairment (PRE vs. POST) was implemented in the model as the independent (fixed) factor. Tinnitus (present vs. absent) and the number of hearing aids (none vs. 1 vs. 2) were introduced as covariates. Psychological measures (BDI, STAI, WHOQOL-BREF, NCIQ) were included in the model as dependent variables that were hypothesized to be affected by the described factors. In addition, correlation analyses were applied to the outcomes of the audiological and psychological tests in the patient group, with an additional evaluation of the associations between the duration of the hearing impairment/age at onset in the postlingual partial deafness, duration of hearing aid use, duration of tinnitus, and various aspects of psychosocial well-being. Men and women were compared with respect to all psychological measures. The distribution of responses to all questionnaires was tested for normality using the Kolmogorov–Smirnov test. Mean scores that were not normally distributed were normalized using the Log10 function. This transformation involved the following scales: BDI, STAI-Trait, WHO-BREF QOL: psychological health, social relationship and environment, NCIQ-self-esteem. All statistical analyses were done with SPSS (version 22).

## Results

### Patients with partial deafness vs. normal hearing individuals

**Fig. 2:**
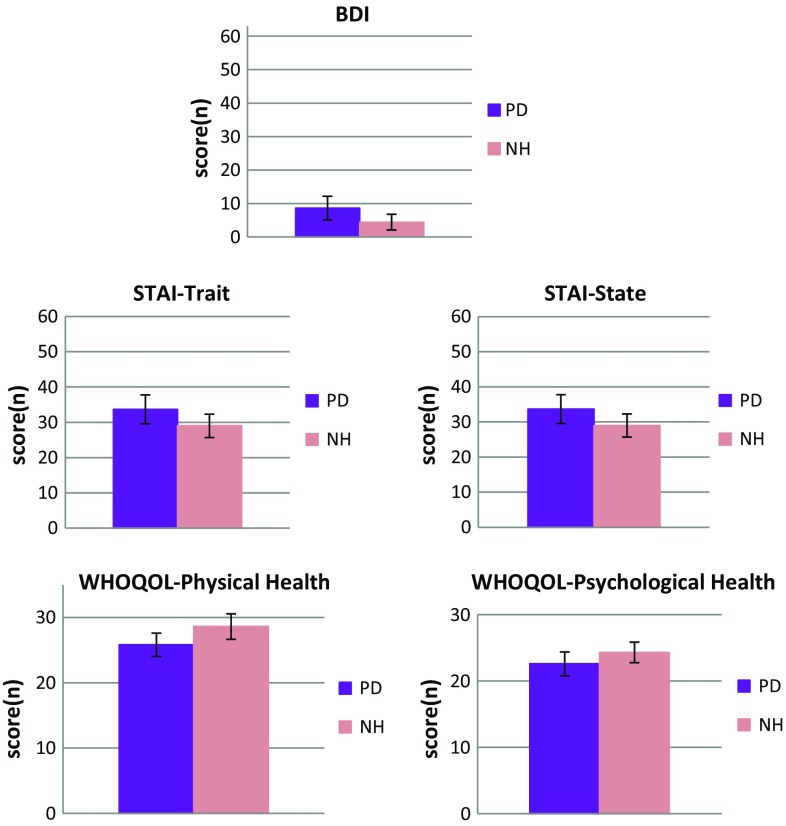
Mean scores obtained by patients and normal hearing individuals in psychological and HRQoL questionnaires that were found different with statistical significance at *p* < 0.05. *Bars* indicate standard deviations. The maximum value on the *y*-axis is the maximum raw score than can be achieved in a particular test. *PD* patients with partial deafness, *NH* normal hearing individuals

PD patients obtained significantly higher scores than the normal hearing group in BDI [*t*(60) = 2.77; *p* = 0.007; PD: *M* = 8.6 ± 7.1; NH: *M* = 4.4 ± 4.7], STAI-State [*t*(60) = 2.49; *p* = 0.016; PD: *M* = 33.7 ± 8.2; NH: *M* = 29.0 ± 6.6] and STAI-Trait [*t*(60) = 2.50; *p* = 0.015; PD: *M* = 33.6, SD = 8.2; NH: *M* = 29.2, SD = 5.5], indicating more psychopathological symptoms in the clinical population. Furthermore, patients had significantly lower scores on the WHOQOL-BREF scales *physical health* [*t*(60) = 2.91; *p* = 0.005; PD: *M* = 25.8 ± 3.6; NH: *M* = 28.6 ± 3.9] and psychological health [*t*(60) = 1.97; *p* = 0.05; PD: *M* = 22.6 ± 3.6; NH: *M* = 24.3 ± 3.1], which suggested decreased health-related quality of life. All results are presented in Fig. 2. From all psychological tools administered to the patient and the normal hearing group, no differences were revealed only for two remaining scales of the WHOQOL-BREF questionnaire, namely the social relationships and the environment subdomains. Scores of men and women were compared in both populations but no statistically significant differences were found (*p* < 0.05).

### Patients with postlingual partial deafness vs. patients with prelingual partial deafness

**Fig. 3:**
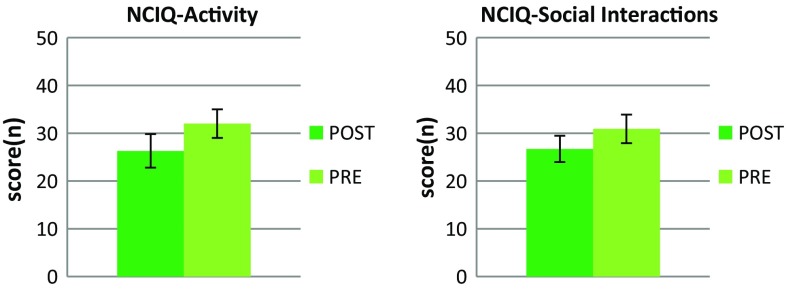
Mean scores on NCIQ scales that provided statistically significant differences between patients with a postlingual and a prelingual onset of partial deafness (*p* < 0.05). *Bars* indicate standard deviations. The maximum value on the *y*-axis is the maximum raw score than can be achieved in a particular test. *POST* patients with postlingual partial deafness, *PRE* patients with prelingual partial deafness

PRE and POST patients obtained comparable mean outcomes in tonal and speech audiometry assessments, as well as hearing aid use and tinnitus (see Table 2). Statistically significant differences between the two clinical subgroups in NCIQ were revealed using GLM. Patients with a prelingual onset of hearing impairment had higher scores on the NCIQ *activity* scale [*F*(1,27) = 4.3; *p* = 0.047; POST: *M* = 26.3 ± 7.0; PRE: *M* = 32.0 ± 7.8] and the NCIQ social interactions scale [*F*(1,27) = 3.7; *p* = 0.050; POST: *M* = 26.7 ± 5.3; PRE: *M* = 30.9 ± 6.0]. Figure 3 depicts the results. No statistically significant between-group differences were demonstrated for BDI, STAI, WHOQOL-BREF and the remaining scales of the NCIQ tool, i.e. basic sound perception, advanced sound perception, speech production, and self-esteem. No impact of tinnitus on HRQoL and mental distress was revealed. However, the applied one-way ANOVA and post hoc tests (Bonferroni corr.) indicated an advantage of non-users over users of one or two hearing aids in audiological tests (averaged for both ears), i.e. PTA, SDT, WRS outcomes, as well as NCIQ subscales assessing communication skills, advanced sound perception and speech production. All statistically significant effects are presented in Table 3.

**Table 3:** Mean scores and comparisons in tests showing statistically significant differences between patients with various hearing aid use; the presented results were significant at p < 0.05

	Non-users (*N* = 12)	Users of one HA (*N* = 10)	Users of two HAs (*N* = 9)	Non-users vs. users of one HA vs. users of two HAs (*F*)	Non-users vs. users of one HA sig.	Non-users vs. users of two HAs sig.
PTA (dB) M(SD)	48.4 ± 17.7	66.1 ± 19.6	81.2 ± 10.4	10.1	0.050	0.000
SDT (dB) M(SD)	44.2 ± 16.9	70.9 ± 16.8	79 ± 8.4	16.1	0.001	0.000
WRS (%) M(SD)	76.2 ± 19.8	50 ± 24.9	41.9 ± 20.4	7.3	0.027	0.000
NCIQ advanced sound perception	38.4 ± 5.1	29.6 ± 6.4	31 ± 6.0	7.4	0.005	0.031
NCIQ speech production	41.9 ± 6.5	33.8 ± 6.1	33.7 ± 5.6	6.6	0.015	0.018

*HA* hearing aid, *PTA* pure-tone average, *SDT* speech detection threshold, *WRS* word recognition score, *M* mean, *SD* standard deviation, *sig.* level of significance in post hoc comparisons, *NCIQ* Nijmegen Cochlear Implant Questionnaire, *dB* decibels

### Correlation analyses

There were statistically significant *r*-Pearson’s correlations revealed between audiological, demographic and psychological measures in patients (jointly POST and PRE) (*p* < 0.05). Higher PTA values averaged for both ears were associated with lower basic and advanced sound perception scores in NCIQ (*r* = −0.37 and *r* = −0.39, respectively). The same correlations were found for SDT (*r* = −0.38 and *r* = −0.44, respectively). In addition, higher SDT averaged for both ears implied lower outcomes on the NCIQ speech production scale. The correlation coefficients revealed for each ear separately were of a similar extent. With a positive correlation apparent for the left-ear WRS and advanced sound perception, there were no significant relationships detected for the right-ear WRS, nor when the scores were averaged for both ears. This was found although WRS scores for the left and the right ear were not statistically different [paired *t* test: *t*(29) = 1.6; *p* = 0.16]. Moreover, with higher PTA patients had also higher SDT (*r* = 0.82) and lower WRS (*r* = −0.82) (averaged for both ears). In addition, lower SDT values were associated with a steeper slope hearing loss (*r* = −0.53). All these effects were present ipsilaterally and contralaterally. A complementary analysis including only patients with a postlingual onset of partial deafness (*N* = 17) produced positive correlations between SDT and partial deafness duration (*r* = 0.53), as well as age at onset (*r* = −0.56). Variables, age at onset of hearing loss and hearing loss duration, were not found to be associated with any other audiological, demographic or psychological outcomes. There were no statistically significant associations detected for the duration of hearing aid use and duration of tinnitus with aspects of quality of life and psychopathology (*p* < 0.05).

## Discussion

The current study provides evidence that patients with partial deafness are a specific population that can experience psychological challenges potentially related to their disability. The cross-sectional design, however, does not permit inferring about causal relationships between the sensory loss and the mental well-being. Literature frequently reports an elevated depressed mood, assessed with depression and anxiety questionnaires in the hearing-impaired population [1–4, 8, 9, 11]. In the present study the effect was also statistically significant but not spectacular, especially with respect to the maximum scores that could be obtained in tests, and suggests low- to medium-range levels of depression and anxiety in both the normal hearing and the patient population (see e.g. work by Leigh and colleagues who used BDI II to evaluate depressive symptoms in hearing deficits) [6]. More sizable effects seen in literature might have been due the fact that the majority of the recruited patients had severe to profound deficits across all frequency ranges, as opposed to partial deafness where the low-frequency hearing loss is mild to moderate. Interestingly, Tambs and colleagues provided vast data on psychopathology in hearing-impaired patients and showed that high and middle frequency hearing abilities affected mental health only to a little extent provided that the low-frequency hearing was within normal ranges (in the current population 90 % had HL < 50 dB HL in the range 0.125–0.5 kHz) [28]. Furthermore, the often reported affective symptoms in patients with prelingual hearing deficits involved patients unfamiliar with spoken language, which might have additionally and considerably affected their mental well-being [3, 4, 11–13]. Nevertheless, during psychological consultations provided at the Institute of Physiology and Pathology of Hearing within the frames of the present study, mood and coping problems have been reported by patients with partial deafness. The authors believe that this population, despite of the preserved low-frequency hearing, might have to face social and emotional challenges that go far beyond those experienced by the normally hearing and which might require professional attention. This especially seems so, as further outcomes of the current study revealed decreased quality of life in patients related to the overall physical and psychological health. This was found despite absence of any additional serious handicaps in the tested population [cf. 3, 7]. Physical health refers to, among other aspects, the required medical care, pain experience, energy level and quality of sleep. One possible reason of lower quality of life among patients might be related to their naturally enhanced focus on health and medical issues which results from their hearing problems. At the same time, however, psychosomatic symptoms might be specifically associated with the auditory impairment which would also be reflected in some patients reporting an elevated depressive mood (with one-third of the BDI questions referring to physical aspects of well-being). The reported significantly deteriorated psychological health, including, among others, the extent of problems with self-esteem, internal coherence, mood and concentration in the patient population lends further support to the latter hypothesis. Similar outcomes were reported by Fellinger and colleagues who also used the WHOQOL-BREF tool to assess health-related quality of life in prelingual signing individuals [11]. The authors pointed to the possible feelings of insecurity and inferiority in deafness when living in a “perfectly” hearing world.

The present trial revealed no significant relationship between the objectively measured severity of the hearing deficit, depressive/anxiety levels and HRQoL, replicating outcomes of several cohort studies [1–3, 7]. The implication would be that mental well-being predominantly depends on the subjective perception of the disability, as well as appropriate medical interventions and not the degree of the impairment itself. Moreover, no effect of tinnitus was demonstrated in patients with partial deafness, which was probably due to the fact that all of them perceived this comorbidity as non-bothersome. As literature shows, it is the level of annoyance with tinnitus that might become a predictor of mental distress in hearing-impaired patients [1–3, 29]. The history of hearing aid use has been recognized by some authors as improving life quality and decreasing psychopathology among patients [7, 30]. We argue, however, that this effect is only true for patients who definitely require and are satisfied with such amplification. Almost 40 % of the patients who participated in the current trial had no benefit from hearing aids and were non-users. This was probably related to their moderate audiometric thresholds, as well as individual features of the auditory system. These patients were actually most proficient in advanced sound perception and speech production, suggesting a significant involvement of residual acoustic hearing in these functions [5, 17]. Should their hearing impairment progress, however, these patients might be re-referred for fitting of hearing aid before a cochlear implantation (e.g. an electro-acoustic system) is considered. At the same time, there was no clear impact demonstrated of the number of hearing aids used (unilateral *vs* bilateral) on any aspect of health/hearing-related quality of life. According to personal communication there were comparable levels of hearing aid satisfaction across all users. Several other studies also failed to show a clear relationship between hearing aid use and mental well-being [2, 3].

There were differences demonstrated between patients with a prelingual onset of hearing loss and those who developed partial deafness during their lifetime with respect to social activity and interactions. The prelingual group showed an advantage. The activity domain refers to the subjectively perceived limitations imposed by the hearing loss on daily professional, family and leisure activities, with social interactions assessing the quality of personal contacts with close family and friends, as well as complete strangers. Patients with prelingual partial deafness due to their residual hearing can never become members of the often stigmatized deaf culture using sign language. Furthermore, due to the lifetime experience of disability, they seem to develop efficient communication modes, as well as coping and adjustment mechanisms [3, 4, 31]. In contrast, patients with an acquired hearing deficit tend to miss fluent audio-verbal communication which they might still recall. It may well be that these patients experience confusion and insecurity, which they yet have to cope with, when devoid of the healthy auditory sense. Consequently, patients with a later onset of hearing loss are more prone to withdraw from social participation [2–4, 31]. At the same time, however, the current analysis failed to demonstrate statistically significant differences between patients with a prelingual and a postlingual onset of hearing impairment with respect to depression/anxiety symptoms, as well as health-related QoL. This finding suggests that other factors should be considered as more predictive than the age at onset of a hearing deficit, especially that both patient populations used spoken language at the time of the study, such as e.g. personal attitude towards the impairment [3].

As to relationships between various measures applied to patients in the present study, there was a clear correlation detected between audiometric thresholds and the subjectively rated sound perception, suggesting a direct relationship between tests performed in a clinical setting and real-life situations related to hearing problems [cf. 27]. Furthermore, an interesting association was revealed for left-ear audiometric word recognition scores only with the subjectively indicated level of recognition of advanced sounds. The authors hypothesize that the effect might be due to the fact that the aspects of auditory processing, such as listening to music and prosody are mainly subserved by the right brain hemisphere, which chiefly receives the information delivered to the left ear (see a review paper by Friederici and Alter [32]). This discussion, however, goes far beyond the scope of this report. These findings seem to confirm the relevance of the Nijmegen-Cochlear-Implant-Questionnaire, as do the significant differences revealed with this tool between patients with a postlingual and a prelingual onset of partial deafness. The instrument had received a very positive feedback from the participating patients who stated that the NCIQ tool focuses on the very basic and fundamental hardships related to their hearing deficits. Similar subdomains of the hearing loss-related quality of life seem to be affected by partial deafness, as is the case of other types of hearing losses [19, 20, 26, 27].

In the patient group there were also several statistically significant correlations established between various audiological measures. Among other outcomes, it has been found that patients with steeper hearing losses in frequency ranges between 0.5 and 2 kHz found it easier to detect speech (lower SDT scores). This effect of hearing loss configuration on speech perception has already been described by Hornsby and colleagues who argued that patients with ski-sloping sensorineural impairments have better ability to use low-pass filtered speech, when compared to their counterparts with flat hearing loss configurations [21]. Listening experience was indicated as one possible explanation of this effect, with another suggesting an enhancement of cortical representations of low-frequency sounds due to high-frequency cochlear dead regions [33]. Interestingly, the correlations were also found for contralateral outcomes, clearly suggesting that listening to speech is a complex binaural phenomenon. Further large-population investigation is required to elucidate the relationship between slope and speech understanding (word recognition scores) which failed to reach statistical significance in this trial. In addition, in patients with postlingual partial deafness worse speech detection thresholds without aiding were related to a longer duration of hearing loss, as well as a younger age at the onset (first use of hearing aids, as reported by the patient). The authors hypothesize that the effect originates in a prolonged exposure to degraded speech, especially with the old-generation hearing aids, whereas some patients should have been earlier equipped with a cochlear implant. Notably, duration of hearing impairment has been suggested as one possible predictor of auditory performance with a cochlear implant [5, 18].

To be able to extrapolate the findings of the study on the entire population of patients with partial deafness, further longitudinal investigation is required before and after cochlear implantation. In addition, considerable care should be taken to provide a precise account of patient’s self-evaluation of hearing aid use and the collateral tinnitus. The authors are aware that some bias could be introduced to the results by the fact that only patients willing to participate and probably those more extrovert decided to participate in the study and complete questionnaires that directly reflect personal attitudes and feelings.

## Conclusions

In the current study well-recognized research instruments used in full self-administration served to provide comprehensive information about mental state and health-related quality of life in patients with partial deafness on high frequencies. NCIQ has proven a valuable tool for the pre-CI assessment in this population but its use in the evaluation of treatment outcomes remains to be confirmed in follow-up studies. The results indicate that auditory rehabilitation has to start early and go beyond training speech in laboratory settings. If necessary, the goal of professionals should be to design personalized treatment programs by following individual psychological needs of these patients for longer periods of time, before and after cochlear implantation. Development of new tools is needed to investigate factors that shape the extent to which a hearing loss is considered a handicap and thus what outcomes are to be expected from treatment with a hearing aid or a cochlear implant. Studies involving the population of patients with partial deafness are of particular value given the ever developing medical solutions allowing the preservation of natural hearing, including new systems combining a cochlear implant and a hearing aid, soft cochlear implant electrode arrays, and soft surgical methods [15, 18]. The perspective of combining the residual hearing of a patient with an artificial aiding system might require a new approach to auditory and psychological rehabilitation.
